# Minimum Adversarial Examples

**DOI:** 10.3390/e24030396

**Published:** 2022-03-12

**Authors:** Zhenyu Du, Fangzheng Liu, Xuehu Yan

**Affiliations:** College of Electronic Engineering, National University of Defense Technology, Hefei 230037, China; liufangzheng17@nudt.edu.cn (F.L.); yanxh17@nudt.edu.cn (X.Y.)

**Keywords:** information security, minimum adversarial examples (AEs), controllable optimization of AEs, *L_p_* constraint, *SSIM* constraint

## Abstract

Deep neural networks in the area of information security are facing a severe threat from adversarial examples (AEs). Existing methods of AE generation use two optimization models: (1) taking the successful attack as the objective function and limiting perturbations as the constraint; (2) taking the minimum of adversarial perturbations as the target and the successful attack as the constraint. These all involve two fundamental problems of AEs: the minimum boundary of constructing the AEs and whether that boundary is reachable. The reachability means whether the AEs of successful attack models exist equal to that boundary. Previous optimization models have no complete answer to the problems. Therefore, in this paper, for the first problem, we propose the definition of the minimum AEs and give the theoretical lower bound of the amplitude of the minimum AEs. For the second problem, we prove that solving the generation of the minimum AEs is an NPC problem, and then based on its computational inaccessibility, we establish a new third optimization model. This model is general and can adapt to any constraint. To verify the model, we devise two specific methods for generating controllable AEs under the widely used distance evaluation standard of adversarial perturbations, namely $L_{p}$ constraint and SSIM constraint (structural similarity). This model limits the amplitude of the AEs, reduces the solution space’s search cost, and is further improved in efficiency. In theory, those AEs generated by the new model which are closer to the actual minimum adversarial boundary overcome the blindness of the adversarial amplitude setting of the existing methods and further improve the attack success rate. In addition, this model can generate accurate AEs with controllable amplitude under different constraints, which is suitable for different application scenarios. In addition, through extensive experiments, they demonstrate a better attack ability under the same constraints as other baseline attacks. For all the datasets we test in the experiment, compared with other baseline methods, the attack success rate of our method is improved by approximately 10%.

## 1. Introduction

With the wide applications of a system based on DNNs, the concerns of their security become a focus. Recently, researchers have found that adding subtle perturbations to the input of deep neural networks causes models to give a wrong output with high confidence. Furthermore, they call the deliberately constructed inputs adversarial examples (AEs). The attack of DNNs by AEs is called adversarial attacks. These low-cost adversarial attacks can severely damage applications based on DNNs. Adding adversarial patches onto traffic signs can lead to auto-driving system error [1]. Adding adversarial logos to the surface of goods can impede automatic check-out in automated retail [2]. Generating adversarial master prints can destroy deep fingerprint identification models [3]. In any of the aforementioned scenarios, AEs can cause great inconvenience and harm people’s lives. Therefore, AEs become an urgent issue in the area of AI security.

In the research on generating AEs, two fundamental problems exist: (1) What is the minimum boundary of the amplitude of adversarial perturbations? All the models try to generate AEs with smaller adversarial perturbations. It is their objective to add as few adversarial perturbations as necessary to the clean example to achieve the attack; (2) Is the minimum boundary of adversarial amplitude reachable? The reachability refers to whether examples with adversarial perturbations that are under a minimum bound of adversarial amplitude can successfully attack as well as whether AEs exist under that boundary.

In order to answer those two problems, traditional AE generation can be devised into two main optimization models: (1) Taking the successful attack as the objective function and the limitation of perturbations as the constraint. This limitation is usually limited as less than or equal to a value, as shown in Equation (1). For a neural network *F*, input distribution $ℵ\subset R^{n}$, a point $X_{0}\in ℵ,X\in R^{n}$, *X* is the adversarial example of $X_{0}$ under the *v* constraint. *D* is the distance metric function:

(1) $$\begin{array}{cc} \; & F\left(X\right)\neq F\left(X_{0}\right)\\ \; & \;s.t.\;D\left(X,X_{0}\right)\leq v \end{array}$$

(2) Taking the minimum of adversarial perturbations as the target and the success of the attack as the constraint:

(2) $$\begin{array}{cc} \; & \min D\left(X,X_{0}\right)\\ \; & \;s.t.\;F\left(X\right)\neq F\left(X_{0}\right) \end{array}$$

However, the above two models do not solve the two problems well: (1) for the first model, when setting the limitation of AEs in the constraint, whether the model has a solution depends on the limit value *v*. The model may have no solution when the limit value *v* is too small. However, when the limit value is larger, the constraint on the AEs is too relaxed, and thus the gap between the solution and the minimum AEs is larger; (2) For the second model, when the limitation of adversarial perturbations is in the objective function, the perturbations will decrease in the whole optimization process until it drops in the local optimum of the whole objective function. This optimization model can easily fall into local optimization so that the solution is not the minimum adversarial example. At the same time, this paper also proves that finding the minimum AEs is an NPC problem, so it cannot find the real minimum AEs.

Therefore, in this paper, we focus on answering the problems mentioned above. For the first problem, we propose the concept of minimum AEs and give the theoretical lower bound of the amplitude of minimum adversarial perturbations. For the second problem, we prove that generating the minimum adversarial example is an NPC problem, which means that the minimum boundary of adversarial amplitude is computationally unreachable. Therefore, we generate the controllable approximation of the minimum AEs. We use the certified lower bound of minimum adversarial distortion to constrain the adversarial perturbations and transform the traditional optimization problem into another new model. (3) Taking the successful attack as a target and the adversarial perturbations are equal to the lower bound of the minimum adversarial distortion plus a controllable approximation, as shown in Equation (3). $\varepsilon_{NNS}$ is the lower bound of the minimum adversarial distortion and $\delta_{\varepsilon }$ is a constant of controllable approximation:

(3) $$\begin{array}{cc} \; & F\left(X\right)\neq F\left(X_{0}\right)\\ \; & \;s.t.\;D\left(X,X_{0}\right)=\varepsilon_{NNS}+\delta_{\varepsilon } \end{array}$$

This model has two advantages compared with the existing methods: (1) Better attack success rate under the same amplitude of adversarial perturbations. Based on the theoretical lower bound of the amplitude of the minimum perturbations, the AEs overcome the blindness of the existing methods by controlling the increment in that amplitude and improve the attack success rate of the AEs. (2) More precisely controlled amplitude of adversarial perturbations under different constraints. The amplitude of the adversarial perturbations will affect the visual quality of AEs. To go a step further, for different scenarios of applications of the AEs, the requirements of visual quality are different. In some scenarios, they are very strict, while others are relaxed. There are two common scenarios as follows: (1) collaborative evaluation of humans and machines. In that case, AEs need to deceive both human oracles and the classifiers based on DNNs. For example, in the scenario of auto-driving, if the patches too easily draw humans’ attention, these adversarial signs would be moved and they would lose their adversarial effect. (2) Single evaluation of machines. In that case, only the classifiers and models based on DNNs need to be bypassed. In the scenario of massive electronic data filtering, they have a low probability of human involvement. When filtering and testing the harmful data involving violence and terrorism, it may heavily depend on the machines so that it has lower requirements for visual quality. Therefore, in order to adapt the two entirely different scenarios, we need to be able to controllably generate AEs.

**Figure 1:**
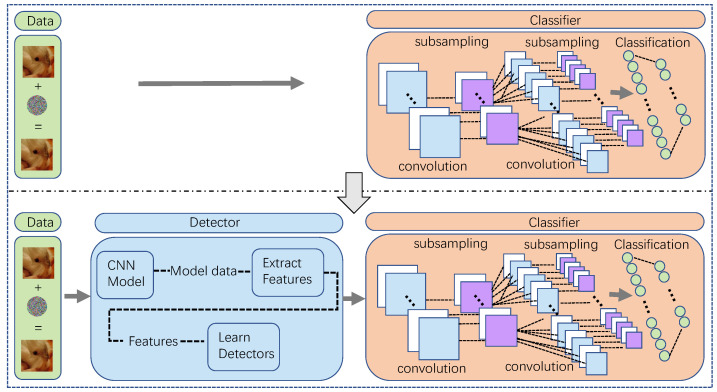
Figure representing the framework of the settings. The top framework is the traditional attack setting and the bottom is our attack setting. In the top setting, the target of the adversarial attack is a single target classifier while our setting is a combined network including a target classifier and a detector.

Meanwhile, generating controllable AEs also brings additional benefits. There are two different views with different implications: (1) Attackers can adaptively and dynamically adjust the amplitude of perturbations. As the described above, the defense technologies against adversarial attacks are mainly detection methods. From the attackers’ point of view, when their target is a combined network or system with detectors in front of the target classifier, as Figure 1 shows, they will expect to evaluate the successful probability of attacking the combined network before implementing the attack. For example, supposing that they know the probability of AEs with fixed perturbation bypassing the detector in advance according to prior knowledge, then they can purposefully generate AEs with bigger perturbations or more minor perturbations with a better visual quality to human eyes. (2) Defenders can actively defend against the attacks with the help of the outputs of controllable AEs. From the defenders’ point of view, controllable AEs can help evaluate defenders’ abilities against the AEs of different modification amplitude. When inputting different AEs with fixed adversarial perturbations to models, the defenders can evaluate their anti-attack capabilities according to the outputs against the unclean examples and then decide whether to add additional defense strategies with an emphasis on the current setting. For the example mentioned in the last point, if the defender has prior knowledge about the attackers’ average perturbation amplitude, they can select whether additional defensive measures are necessary.

In this paper, we first give the definitions of minimum adversarial perturbations and AEs and the theorem of generating minimum AEs as an NPC problem and then propose a new model of generating adversarial examples. Furthermore, we give two algorithms for generating an approximation of AEs under $L_{p}$ and SSIM constraints. We perform experiments under widely used datasets and models for all the datasets tested in the experiment; compared with other baseline methods, the attack success rate of our method is improved by approximately 10%.

Our contributions are as follows:

- We first prove that generating minimum AEs is an NPC problem. We then analyze the existence of AEs with the help of the definition of the lower bound of the minimum adversarial perturbations. According to the analysis, we propose a general framework to generate an approximation of the minimum AEs.
- We propose the methods of generating AEs with a controllable amplitude of AEs under the $L_{2}$ and SSIM constraints. Additionally, we further improve the visual quality in case of greater perturbations.
- The experiments demonstrate that our method has a better performance in terms of attack success rate than other widely used methods at baseline under the same constraint. Meanwhile, its performance of precisely controlled amplitude of adversarial perturbations under different constraints is also better.

The rest of this paper is organized as follows. In Section 2, we briefly review the related work. In Section 3, we describe the basic definition, theorem and model of our algorithm in detail and prove the theorem. In Section 4, we give the transformed model of the basic model under two constraints and provide the efficient solution algorithm of the two models, respectively, in the two subsections. In Section 6, we present our experimental results and compare them with other baseline methods. Finally, we conclude our paper in Section 7.

## 2. Related Work

### 2.1. Adversarial Attack

There are two main pursuits of AEs: one is the smaller perturbations of the AEs; and the other is the successful attack. Previous works transform the two pursuits into two main optimization models. One takes the successful attack as the objective function and the limitation of perturbations as the constraint. These works include L-BFGS [4], C&W [5], DF [6] and HCA [7]. The other takes the successful attack as the objective function and the limitation of perturbations as the constraint. Such works include UAP [8], BPDA [9] and SA [10]. Other works, including FGSM [11], JSMA [12], BIM [13] and PGD [14] do not directly use the model of the optimization problem. However, these methods convert the successful attack into a loss function, move it along the direction of the decrease or increase in the loss function to find the AEs, and use a value at each step to constrain the perturbations. They can be classified as the second optimization model from the point of method-based view.

However, these works cannot really find the minimum AEs with the minimum amplitude of adversarial perturbations. For the first model, the model may have no solution when the value is set as too small. Furthermore, for the second model, it is easy to fall into local optimization.

Meanwhile, considering the constraint function of adversarial perturbations, the works of adversarial example generation can be divided into two main classes. One AEs generation under $L_{p}$ constraint, including $L_{0}$ constraint [14,15], $L_{2}$ constraint [14] and $L_{\infty }$ constraint [11,13,14], which is widely used. Furthermore, in addition to that $L_{p}$ constraint, there were other constraints in previous studies. In [16], the authors proposed that the commonly used $L_{p}$ constraint failed to completely capture the perceptual quality of AEs in the field of image classification. This used the structural similarity index SSIM [17] measure to replace that constraint. Moreover, the other two works [18,19] also used perceptual distance measures to generate AEs. The work [18] used SSIM while [19] used the perceptual color distance to achieve the same purpose.

However, the constraint of those works is not strict. For the AEs generation under the $L_{p}$ constraint, it is hard to control the amplitude of perturbations and there is a deviation of AEs generated by those works. For the other constraints, they cannot strictly control the perceptual visual quality: neither the SSIM value nor perceptual color distance.

Therefore, in this paper, we search for the minimum AEs with the minimum amplitude of perturbations. Moreover, we prove that generating the minimum AEs is an NPC problem. Furthermore, we transform that problem into the new optimization model that generates the controllable approximation of the minimum AEs. We generate AEs with a controllable amplitude of adversarial perturbations under the $L_{p}$ constraint and SSIM constraint, respectively.

### 2.2. Certified Robustness

The robustness of neural networks focuses on searching the lower bound and upper bound of the robustness of neural networks. The lower bound of the robustness is that there are no AEs when adding adversarial perturbations that are less than or equal to that boundary. Moreover, the upper bound of the robustness adding AEs that are larger than or equal to that bound can always acquire the AEs. The work CLEVER [20] and CLEVER++ [21] were the first neural network robustness evaluation scores. They use extreme value theory to estimate the Lipschitz constant based on sampling. However, that estimation requires many samples to have a better value of estimation. Therefore, the two methods only estimate the lower bound of the robustness of neural networks and cannot provide certification. As follows, the works Fast-Lin and Fast-Lip [22], CROWN [23] and CNN-Cert [24] are methods of certifying the robustness of the neural networks. The Fast-Lin and Fast-Lip [22] can only be used for neural networks with the activation function of ReLu. CROWN [23] can be further used for the networks with all general activation functions. Furthermore, the CNN-Cert [24] can be used for the general convolutional neural networks (CNNs). The basic idea is constructing linear functions to constrain the input and then using the upper and lower bounds of the functions as the upper and lower bounds of input, respectively. After that, it can constrain the whole network layer by layer. The whole process is iterative.

However, the above algorithm does not indicate how to calculate the AEs according to the calculated lower bound, and the reachability of AEs based on the lower bound remains a problem. Therefore, in this paper, we calculate the approximation of the minimum AEs based on the lower bound.

## 3. Basic Definition, Theorem and Modeling

**Definition** **1.** *(AEs, Adversarial Perturbations). Given a neural network F, a distribution $ℵ\subset R^{n}$, a distance measurement $D:R^{n}\times R^{n}\to R$ between X and $X_{0}$, a point $X_{0}\in ℵ$ and a point $X\in R^{n}$, we say that X is an adversarial example of $X_{0}$ under constraint $\varepsilon_{0}$ if $F\left(X\right)\neq F\left(X_{0}\right)$ and $D\left(X,X_{0}\right)=\varepsilon_{0}$.*

**Definition** **2.** *(Minimum AEs, Minimum Adversarial Perturbations). Given a neural network F, a distribution $ℵ\subset R^{n}$, a distance measurement $D:R^{n}\times R^{n}\to R$ between X and $X_{0}$, and a point $X_{0}\in ℵ$, we say that $X^{*}\in R^{n}$ is a minimum adversarial example of $X_{0}$ if $X^{*}$ is an adversarial example of $X_{0}$ under constraint $\varepsilon^{*}$ and $\varepsilon^{*}=\min_{X}\varepsilon_{0}$ such that there exists an adversarial example of $X_{0}$ under constraint $\varepsilon_{0}$. $\varepsilon^{*}$ is the minimum adversarial perturbations of $X_{0}$ under D constraint.*

**Theorem** **1.** *Given a neural network F, a distribution $ℵ\subset R^{n}$, a distance measurement $D:R^{n}\times R^{n}\to R$ between X and $X_{0}$ and a point $X_{0}\in ℵ$, searching for a minimum adversarial example of $X_{0}$ is an NPC problem.*

**Proof.**  The proof of Theorem 1 is shown in Appendix A. □

Although it is an NPC problem, researchers calculate the non-trivial upper bounds of the robustness of the neural network [23,24,25]. We can thus calculate the non-trivial lower bounds of the minimum adversarial perturbations $\varepsilon_{NNS}$ of $X_{0}$ based on the exact meaning of the two bounds.

We thus model the problem of calculating the non-trivial lower bounds of the minimum adversarial perturbations $\varepsilon_{NNS}$ of $X_{0}$. For input distribution $ℵ\subset R^{n}$, a clean input $X_{0}$, perturbed input *X* of $X_{0}$ under the ε constraint, $X\in B\left(X_{0},\varepsilon \right)$, $B=\left\{X:D(X,X_{0})\leq \varepsilon \right\}$, a neural network $F:R^{n}\to R^{k}$, original label *y* of $X_{0}$, $F(X_{0})=y$, target label $y^{*}$, $y^{*}\neq y$, and we define the non-trivial lower bounds of the minimum adversarial perturbations as $\varepsilon_{NNS}$ of $X_{0}$, as shown in Equation (4):

(4) $$\varepsilon_{NNS}=\max_{y^{*}\neq y}\varepsilon_{y^{*}}^{*}$$

and:

(5) $$\begin{array}{c} \varepsilon_{y^{*}}^{*}=\min\epsilon \\ \;s.t.\;\gamma_{y}^{U}\left(X\right)-\gamma_{y^{*}}^{L}\left(X\right)\leq 0 \end{array}$$

In Equation (4), $\varepsilon_{y*}^{*}$ is the minimum of adversarial perturbations of $X_{0}$ under the target label $y^{*}$. In Equation (5), ε is the perturbation of $X_{0}$ such that $F\left(X\right)=y^{*}$, $\gamma_{y}^{U}\left(X\right)$ means the upper bound of the network under label *y* of input *X* and $\gamma_{y^{*}}^{L}$ means the lower bound of the network under another label $y^{*}$ of the input. They are calculated in [23,24,25].

**Theorem** **2.** *Given a neural network F, a distribution $ℵ\subset R^{n}$, a distance measurement $D:R^{n}\times R^{n}\to R$ between X and $X_{0}$, a point $X_{0}\in R^{n}$, the non-trivial lower bounds $\varepsilon_{NNS}\in R$ of the minimum adversarial perturbations of $X_{0}$, if X is the perturbed example of $X_{0}$ under constraint $\varepsilon_{NNS}$ and $X\in B(X_{0},\varepsilon_{NNS})$, then $F\left(X\right)\equiv F\left(X_{0}\right)$.*

**Proof.**  According to the definition and meaning of the $\varepsilon_{NNS}$, we can obtain Theorem 2. □

**Definition** **3.** *(N-order tensor [26]). In deep learning, a tensor extends from a vector or matrix to a higher dimensional space. The tensor can be defined by a multi-dimensional array. The dimension of a tensor is also called order, that is, N-dimensional tensor, also known as N-order tensor. For example, when N=0, the tensor is a 0-order tensor, which is one number. When N=1, the tensor is a 1-order tensor, which is a 1-dimensional array. When N=2, the tensor is a 2-order tensor, which is a matrix.*

**Definition** **4.** *(Hadamard product [26]). The Hadamard product is the element-wise matrix product. Given the N-order tensors $A,B\in R^{I_{1}\times I_{2}\times ...\times I_{N}}$, the Hadamard product A×B is denoted as the product of elements corresponding to the same position of the tensor. The product C is a tensor with the same order and size as A and B. That is:*

(6) $$C=A\times B,C_{i_{1},\dot{2},\ldots ,i_{n}}=A_{1,i_{2},\ldots ,i_{n}}\times B_{i_{1},i_{2},\ldots ,i_{n}}$$

**Definition** **5.** *(${+}^{*}$). For a real number λ∈R and N-order tensor X$\in R^{I_{1}\times I_{2}\times ...\times I_{N}}$, we define $\lambda {+}^{*}X$ as the sum of X and the Hadamard product of λ and another tensor $\Psi \in R^{I_{1}\times I_{2}\times ...\times I_{N}}$. That is:*

(7) $$D=\lambda {+}^{*}X=\lambda \times \Psi +X,\;D_{i_{1},i_{2},\ldots ,i_{n}}=\lambda \times \Psi_{i_{1},i_{2},\ldots ,i_{n}}+X_{i_{1},i_{2},\ldots ,i_{n}}$$

*D, Ψ and X have the same order. Specifically, in the field of the AEs, given a clean input $X_{0}\in R^{N}$, and perturbations r∈R, the adversarial example is $X=X_{0}{+}^{*}r=X_{0}+\Psi \times r$. The physical meaning is the proportionality factor of r which adds on each feature ${X_{0}}_{i_{1},i_{2},...,i_{n}}$.*

*For example, λ=2, $X=\left[\begin{array}{cc} 1 & 2\\ 3 & 4 \end{array}\right]$, $\Psi =\left[\begin{array}{cc} 2 & 3\\ 5 & 6 \end{array}\right]$,*

$D=\lambda {+}^{*}X=\lambda \times \Psi +X=2\times \left[\begin{array}{cc} 1 & 2\\ 3 & 4 \end{array}\right]+\left[\begin{array}{cc} 2 & 3\\ 5 & 6 \end{array}\right]=\left[\begin{array}{cc} 4 & 7\\ 11 & 14 \end{array}\right]$

**Definition** **6.** *(ε∼τ approximation of minimum AEs, ε∼τ approximation of minimum adversarial perturbations). Given a neural network F, a distribution $ℵ\subset R^{n}$, a point $X_{0}\in ℵ$, the non-trivial lower bounds $\varepsilon_{NNS}\in R$ of the minimum adversarial perturbations of $X_{0}$, a constraint $\varepsilon_{\tau }^{*}$ and $\varepsilon_{\tau }^{*}=\varepsilon_{NNS}+\tau$, τ>0 is a constant, we say that $X_{\tau }^{*}$ is the ε∼τ approximation of minimum AEs of $X_{0}$ and $\varepsilon_{\tau }^{*}$ is the ε∼τ approximation of minimum adversarial perturbations such that $X_{\tau }^{*}=X_{0}{+}^{*}\varepsilon_{\tau }^{*}$, $F\left(X_{\tau }^{*}\right)=F\left(X_{0}{+}^{*}\varepsilon_{\tau }^{*}\right)\neq F\left(X_{0}\right)$.*

τ is a constant set by humans according to the actual statement. When generating an adversarial example for a specific input, it has different requirements of the adversarial perturbations for different settings, scenarios and samples.

(a) The more complex the scenario is, the smaller the constant τ is. In the extreme scenario of digital AEs generation, it needs a clear filter of the AEs and has a strict requirement of invisibility, and the τ should be small [15]. However, for most physical AEs generations, it has the relaxed requirement of invisibility. Most of them only need to keep semantic consistency. The τ can be set more considerably than the digital setting [27].

(b) The more simple the sample is, the smaller the constant τ is. When the sample is simple, its information is single, and people would be more sensitive to the perturbations than complex samples. It is easier for people to recognize the difference between clean inputs and perturbed inputs. For example, the τ of the MNIST dataset [28] should be smaller than the CIFAR-10 dataset [29].

We model the problem of generating AEs under *D* measure metrics as follows. For a deep neural network *F*, input distribution $ℵ\subset R^{n}$, a point $X_{0}\in ℵ$ and given the distance value *d* under constraint *D*, the problem of generating controllable AEs of *d* can be modeled as

(8) $$\begin{array}{c} F\left(X\right)\neq F\left(X_{0}\right)\\ \;s.t.\;X\in B=\left\{X:D(X,X_{0})=d\right\} \end{array}$$

We discuss the problem under two settings. One is the constraint of the $L_{p}$ norm, and the other is that of perceptually constrained *D* measure metrics. We use the widely used structural similarity (SSIM) as the perceptual constraint in a perceptually constrained AEs generation. The two constraints will be discussed, respectively, in the following sections.

## 4. AEs Generation under $L_{p}$ Constraint

### 4.1. Analysis of the Existence of AEs

**Figure 2:**
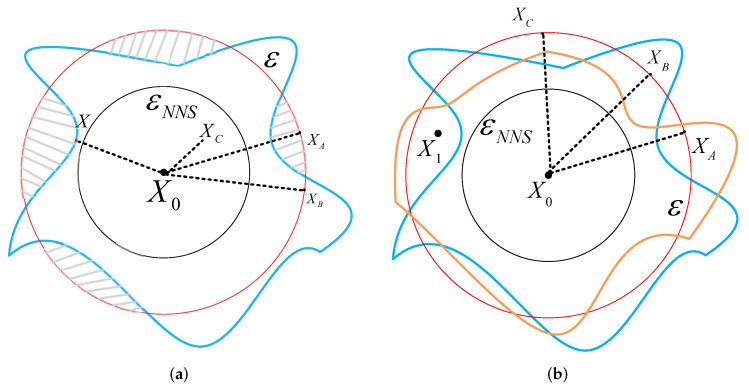
Figure representing the spaces where AEs exist. The black circle indicates the ball of the non-trivial lower bounds of the minimum adversarial perturbations of $X_{0}$; the blue line indicates the classification bound of the network when $X_{0}$ is input; and the red circle means the ball of adding perturbations ε on $X_{0}$. When examples are inside the blue line, they can be classified as the original label by the network. However, when they are outside the blue line, they are AEs. The gray shadow indicates the space where AEs exist under the ball of the ε. The yellow boundary is another classification border of $X_{1}$.

According to Theorem 2, we reach the following conclusions concerning the existence of AEs, as shown in Figure 2.

As Figure 2a shows, we have the following analysis. When adding adversarial perturbations lower than $\varepsilon_{NNS}$, no AEs of $X_{0}$ exist.

When adding adversarial perturbations larger than $\varepsilon_{NNS}$, AEs of $X_{0}$ exist. The gray shadow between the red circle and the blue line is the space where AEs exist. However, whether AEs can be found depends on the direction of adding ε perturbations. As the figure shows, the perturbations of $X_{A}$ and $X_{B}$ all equal ε, and they are all located on the bound of the ball of ε; however, we can see that $X_{A}$ is inside the gray shadow while $X_{B}$ is not.

Therefore, some conclusions that were previously well known hypotheses can be proven. Different AEs generation methods generate AEs with varying accuracy. For a clean input $X_{0}$, when adding the same perturbations on it, method *A* can acquire the adversarial input $X_{A}$. In contrast, method *B* obtains $X_{B}$ located inside the blue line and can still be correctly classified by the network. Hence, the key to generating AEs is finding the direction of where AEs exist. As shown in Figure 2a, when it is along the path of *X*, the added perturbations are the smallest.

Meanwhile, for different clean samples, the added perturbations of generating AEs are different. When a specific perturbation $\varepsilon >\varepsilon_{NNS}$ is fixed, different clean samples will obtain different perturbed examples after adding those perturbations. As shown in Figure 2b, the blue boundary and the yellow boundary are the different classification boundaries of two different samples, respectively. The perturbed examples acquired by adding the same perturbations ε are within the yellow boundary but outside the blue border. Therefore, they are the AEs of the blue boundary constraint samples which can be correctly classified by the yellow boundary constraints. Thus, the adversarial example needs to be researched for a specific sample.

Therefore, according to the analysis of the existence of AEs, we have the following conclusions. In order to generate practical AEs, the added perturbations quantity needs to meet the requirement $\varepsilon >\varepsilon_{NNS}$ and it needs to be larger than the classification boundary in this direction. At the same time, due to the limitation of invisibility of the AEs, it should be as small as possible. Thus, the generation direction of the AEs should be closer to the direction of minimum AEs.

According to Theorem 1, searching for the minimum AEs of sample $X_{0}$ is an NPC problem. In this paper, we try to generate the minimum AEs under a ε∼τ numerical approximation as Definition 6.

According to Figure 2a, in order to generate an effective adversarial example, the perturbations should be larger than the lower bound $\varepsilon_{NNS}$ and the perturbations needed to cross the boundary of the classifier. When fixing $\varepsilon_{\tau }^{*}$, it defines a ball with a center of $X_{0}$ and radius $\varepsilon_{\tau }^{*}$. As shown in Figure 2a, the points on the ball are not all AEs. Using the + method is the same as selecting a random direction to generate perturbed examples that are highly unlikely to be adversarial. Therefore, it is necessary to calculate the direction of adding $\varepsilon_{\tau }^{*}$ and make $F\left(X_{\tau }^{*}\right)=F\left(X_{0}+\Psi \times \varepsilon_{\tau }^{*}\right)\neq F\left(X_{0}\right)$. Ψ is the direct tensor of effective AEs.

### 4.2. Model of $L_{p}$ Constraint

We model the problem of generating the ε∼τ approximation of minimum AEs. For a neural network *F*, the input distribution $ℵ\subset R^{n}$, a point $X_{0}\in ℵ$ and given the ε∼τ approximation of minimum adversarial perturbations $\varepsilon_{\tau }^{*}$, the problem of generating the ε∼τ approximation of the minimum adversarial example $X_{\tau }^{*}$ can be modeled as

(9) $$\begin{array}{c} F\left(X_{\tau }^{*}\right)\neq F\left(X_{0}\right)\\ \;s.t.\;X_{\tau }^{*}\in B_{p}=\left\{X:{∥X-X_{0}∥}_{p}=\varepsilon_{\tau }^{*}\right\} \end{array}$$

According to the analysis of the existence of AEs and Theorem 2, when the added adversarial perturbations $\varepsilon >\varepsilon_{NNS}$, AEs certainly exist and the model must have a solution.

### 4.3. Framework of AE Generation under $L_{p}$ Constraint

According to Definition 6, we transform the problem of calculating the ε∼τ approximation of minimum adversarial example ${X_{0}}_{\tau }^{*}$ into searching for the direct tensor Ψ.

For a neural network *F*, input distribution $ℵ\subset R^{n}$, a point $X_{0}\in ℵ$ and given the ε∼τ approximation of minimum adversarial perturbations $\varepsilon_{\tau }^{*}$, according to Definition 5, the ε∼τ approximation of the minimum adversarial example is $X_{\tau }^{*}$, and $X_{\tau }^{*}=X_{0}{+}^{*}\varepsilon_{\tau }^{*}=X_{0}+\varepsilon_{\tau }^{*}\times \Psi$, which means:

(10) $${∥X_{0}+\varepsilon_{\tau }^{*}\times \Psi -X_{0}∥}_{p}=\varepsilon_{\tau }^{*}$$

(11) $$F\left(X_{0}+\varepsilon_{\tau }^{*}\times \Psi \right)\neq F\left(X_{0}\right)$$

This model must have solutions, and we can consider a special solution. We set one element of $\Psi \in R^{I_{1}\times I_{2}\times \ldots \times I_{N}}$ as 1 and the others are 0, fulfilling Equation (10). When the clean input is an image, it means modifying one channel of one pixel of the image, as proposed in [15]. However, this attack only has a 20.61% success rate on VGG-16 [30] of cifar-10 [29]. Furthermore, the perturbations of this pixel are too large to be set as τ.

It is difficult to directly calculate Ψ; thus, to solve Equation (10), we decompose $\varepsilon_{\tau }^{*}\times \Psi$ into the two tensors δ×Λ and $\delta ,\Lambda \in R^{I_{1}\times I_{2}\times ...\times I_{N}}$, and each element of δ and Λ are defined as $\delta_{i_{1},i_{2},\ldots ,i_{n}}$ and $\Lambda_{i_{1},i_{2},\ldots ,i_{n}}$, respectively. The *n*-order tensor δ determines the location of the added perturbations and the importance of the target label while the *n*-order tensor Λ determines the size of the added perturbations, that is, the percentage of the total perturbations.

According to Equation (10), we obtain the following derivation:

(12) $$\begin{array}{cc} ∥X_{0}+\varepsilon_{\tau }^{*}\times \Psi -X_{0} & ∥{}_{p}=∥\varepsilon_{\tau }^{*}{\times \Psi ∥}_{p}\\ \; & =\sqrt[{p}]{{\left(\varepsilon_{\tau }^{*}\times \Psi \right)}^{p}}\\ \; & =\sqrt[{p}]{{\left(\delta \times \Lambda \right)}^{p}}\\ \; & =p\sqrt{\sum_{i}^{I_{1}}\sum_{i_{2}}^{I_{2}}\ldots \sum_{i.}^{I_{n}}\delta_{i_{1}i_{2}\ldots i_{n}}^{p}\times \Lambda_{i_{1}i_{2}\ldots i_{n}}^{p}}\\ \; & =\varepsilon_{\tau }^{*} \end{array}$$

Therefore:

(13) $$\sum_{i}^{I_{1}}\sum_{i_{2}}^{I_{2}}\ldots \sum_{i.}^{I_{n}}\delta_{i_{1}i_{2}\ldots i_{n}}^{p}\times \Lambda_{i_{1}i_{2}\ldots i_{n}}^{p}={\varepsilon_{\tau }^{*}}^{p}$$

However, in Equation (13), the two tensors are all unknown and all of them have *n* elements, so it is a multivariate *n*-order equation and still unsolvable. Although it is unsolvable, we can certify it as a trivial solution. We can certify when:

(14) $$\Lambda_{i_{1}i_{2}\ldots i_{n}}^{p}={\varepsilon_{\tau }^{*}}^{p}/\sum_{i_{1}}^{I_{1}}\sum_{i_{2}}^{I_{2}}\ldots \sum_{i_{n}}^{I_{n}}\delta_{i_{1}i_{2}\ldots i_{n}}^{p}$$

Equation (14) is workable. The proof is shown as follows.

**Proof.**  (15) $$\begin{array}{cc} \; & \sum_{i_{1}}^{I_{1}}\sum_{i_{2}}^{I_{2}}\ldots \sum_{i_{n}}^{I_{n}}\delta_{i_{1}i_{2}\ldots i_{n}}^{p}\times \Lambda_{i_{1}i_{2}\ldots i_{n}}^{p}\\ \; & =\delta_{11\ldots 1}^{p}\times \Lambda_{11\ldots 1}^{p}+\delta_{11\ldots 2}^{p}\times \Lambda_{11\ldots 2}^{p}+\ldots +\delta_{I_{1}\times I_{2}\times \ldots \times I_{N}}^{p}\times \Lambda_{I_{1}\times I_{2}\times \ldots \times I_{N}}^{p}\\ \; & =\frac{{\varepsilon_{\tau }^{*}}^{p}\times \delta_{11\ldots 1}^{p}}{\sum_{i_{1}}^{I_{1}}\sum_{i_{2}}^{I_{2}}\ldots \sum_{i_{n}}^{I_{n}}\delta_{i_{1}i_{2}\ldots i_{n}}^{p}}+\frac{{\varepsilon_{\tau }^{*}}^{p}\times \delta_{11\ldots 2}^{p}}{\sum_{i_{1}}^{I_{1}}\sum_{i_{2}}^{I_{2}}\ldots \sum_{i_{n}}^{I_{n}}\delta_{i_{1}i_{2}\ldots i_{n}}^{p}}+\ldots +\frac{{\varepsilon_{\tau }^{*}}^{p}\times \delta_{I_{1}\times I_{2}\times \ldots \times I_{N}}^{p}}{\sum_{i_{1}}^{I_{1}}\sum_{i_{2}}^{I_{2}}\ldots \sum_{i_{n}}^{I_{n}}\delta_{i_{1}i_{2}\ldots i_{n}}^{p}}\\ \; & ={\varepsilon_{\tau }^{*}}^{p} \end{array}$$

□

We only need to search for one solution of the model (9). That is, we only need to generate one ε∼τ approximation of a minimum adversarial example corresponding to the requirements (9). The trivial solution Equation (14) is therefore the result.

Therefore, the problem of generating the ε∼τ approximation of minimum AEs is transformed into generating the tensor Ψ by Definition 6 and it is then transformed into calculating the two tensors δ,Λ by Equation (13). Moreover, it is finally transformed into calculating the tensor δ. However, it is still an unsolvable question. Although the only thing we need to do is calculate the tensor δ, it is an *n*-order tensor in the real world so that there are *n* elements that remain unknown and need to be calculated. According to Equation (13), when tensor δ is known, the problem of solving the multivariate *n*-order equation is turned into a multivariate 1-order equation. If we want to solve the multivariate 1-order equation, we need *n* equations. However, we only have one equation, which is Equation (13). Therefore, this paper proposes the solution framework for generating the ε∼τ approximation of minimum AEs and a heuristic method to solve the problem.

### 4.4. Method of Generating Controllable AEs under $L_{p}$ Constraint

According to the definition of the AEs, we decompose the tensor δ into ϖ+αξ, $\varpi ,\xi \in R^{I_{1}\times I_{2}\times ...\times I_{N}}$. Each element of ϖ and ξ are defined as $\varpi_{i_{1},i_{2},\ldots ,i_{n}}$ and $\xi_{i_{1},i_{2},\ldots ,i_{n}}$, respectively.

(16) $$\begin{array}{c} \delta_{i_{1},i_{2},\ldots ,i_{n}}=\varpi_{i_{1},i_{2},\ldots ,i_{n}}+\alpha \xi_{i_{1},i_{2},\ldots ,i_{n}} \end{array}$$

Because the *N*-order tensor determines the position of adding perturbations and the importance of the position to the target label, it contains two factors that restrict the value of the AEs. One is to improve the invisibility of the AEs so that added perturbations should be insensitive to human eyes. Another is to improve the effectiveness of the AEs so that the added perturbations should be able to push the sample away from the original classification boundary (in the case of non-target attack) or close to the target classification boundary (in the case of target attack. Obtaining a balance between the two factors is a key problem in the study of AEs. Therefore, we decompose the δ into ϖ and ξ.

Importantly, ϖ is the tensor to determine the effectiveness of AEs and ξ is the tensor to determine the invisibility of AEs. According to Equation (13), we have:

(17) $$\begin{array}{cc} {\varepsilon_{\xi }^{*}}_{i_{1},i_{2},\ldots i_{n}} & \times \Psi_{i_{1},i_{2},\ldots ,i_{n}}\\ \; & =\left(\alpha_{1}\varpi_{i_{1},i_{2},\ldots ,i_{n}}+\alpha_{2}\xi_{i_{1},i_{2},\ldots ,i_{n}}\right)\times {\varepsilon_{\xi }^{*}}^{p}/\sum_{i_{1}}^{I_{1}}\sum_{i_{2}}^{I_{2}}\ldots \sum_{i_{n}}^{I_{n}}\delta_{i_{1}i_{2}\ldots i_{n}}^{p}\\ \; & =\left(\alpha_{1}\varpi_{i_{1},i_{2},\ldots ,i_{n}}+\alpha_{2}\xi_{i_{1},i_{2},\ldots ,i_{n}}\right)\frac{{\varepsilon_{\xi }^{*}}^{p}}{\sum_{i_{1}}^{I_{1}}\sum_{i_{2}}^{I_{2}}\ldots \sum_{i_{n}}^{I_{n}}\delta_{i_{1}i_{2}\ldots i_{n}}^{p}} \end{array}$$

Therefore, the perturbations added on each element are:

(18) $$\begin{array}{c} X_{i_{1},i_{2},\ldots i_{n}}\\ ={X_{0}}_{i_{1},i_{2},\ldots i_{n}}+\left(\alpha_{1}\varpi_{i_{1},i_{2},\ldots ,i_{n}}+\alpha_{2}\xi_{i_{1},i_{2},\ldots ,i_{n}}\right)\frac{\varepsilon^{p}}{\sum_{i_{1}}^{I_{1}}\sum_{i_{2}}^{I_{2}}\ldots \sum_{i_{n}}^{I_{n}}{\left(\alpha_{1}\varpi_{{\hat{h}}_{1}i_{2},\ldots ,i_{n}}+\alpha_{2}\xi_{{\dot{h}}_{1},i_{2},\ldots ,i_{n}}\right)}^{p}} \end{array}$$

According to the above analysis, we transform the ε∼τ approximation of minimum AEs generation into calculating the ϖ and ξ.

#### 4.4.1. Calculating ϖ

According to the analysis of Equation (5), when $\gamma_{y}^{U}\left(X\right)$ is lower than $\gamma_{y^{*}}^{L}\left(X\right)$, the input *X* is an adversarial example. This means that the upper bound of the network under the original label of input *X* is lower than the lower bound of the network under other labels. Therefore, we let:

(19) $$\begin{array}{c} \varpi_{i_{1},i_{2},\ldots ,i_{n}}=-\nabla_{X}\hbar \left(X\right)\\ \hbar \left(X\right)=\gamma_{y}^{U}\left(X\right)-\gamma_{y^{*}}^{L}\left(X\right) \end{array}$$

In the initial update step, the perturbed examples are not in the shadow space so that they are still correctly recognized by the model and $\gamma_{y}^{U}\left(X\right)-\gamma_{y^{*}}^{L}\left(X\right)>0$. At this time, we need to make the examples as close as possible to reducing the ℏ(X), so the update direction is opposite to the gradient. When the ℏ(X) value is less than 0, the absolute value of the ℏ(X) needs to be larger, but the real ℏ(X) value still needs to decrease so that the update direction remains the opposite of the gradient direction.

#### 4.4.2. Calculating ξ

According to the definition of ξ, ξ is the tensor to determine the invisibility of AEs. DCT transformation [31] can transform the data from host space to frequency domain space, and the data in the time-domain or space-domain can be transformed into a frequency-domain that is easy to analyze and process. When data are image data, after transformation, much crucial visual information about the images is concentrated in a small part of the coefficient of DCT transformation. The high-frequency signal corresponds to the non-smooth region in the image, while the low-frequency signal corresponds to the smoother region in the image.

According to the human visual system (HVS) [17], (1) human eyes are more sensitive to the noise of the smooth area of the image than the noise of the non-smooth area or the texture area; (2) human eyes are more sensitive to the edge information of the image and the information is easily affected by external noise.

Therefore, according to the definition of DCT, we can distinguish the features of each region of the image and selectively add perturbations. Given that the *N*-order tensor input data $X_{0}\in R^{I_{1}\times I_{2}\times \ldots \times I_{N}}$ can be seen as a superposition of $I_{1}\times I_{2}\times \ldots \times I_{N}/I_{i}\times I_{j}$ two-order tensor $X_{0}^{\Pi }\in R^{I_{i}\times I_{j}}$:

(20) $$DCT{\left(X_{0}^{\Pi }\right)}_{k,l}=\frac{2}{\sqrt{i_{i}i_{j}}}c\left(k\right)c\left(l\right)\sum_{m=0}^{i_{i}-1}\sum_{n=0}^{i_{j}-1}X_{0}^{\Pi }{}_{i_{i},i_{j}}\cos\frac{(2m+1)k\pi }{2i_{i}}\cos\left(\frac{(2n+1)l\pi }{2i_{j}}\right)$$

and $m,k\in \left\{0,1,\ldots ,i_{i}-1\right\},n,l\in \left\{0,1,\ldots ,i_{j}-1\right\}$.

(21) $$c\left(k\right)=\left\{\begin{array}{cc} 1/\sqrt{2} & k=0\\ 1 & k=1,2,\ldots ,i_{i}-1 \end{array},\right.c\left(l\right)=\left\{\begin{array}{cc} 1/\sqrt{2} & l=0\\ 1 & l=1,2,\ldots ,i_{j}-1 \end{array}\right.$$

In this paper, according to the definition of the tensor of ξ:

(22) $$\xi_{i_{1},i_{2},\ldots ,i_{n}}=\mathrm{DCT}{\left(X_{0}^{\Pi }\right)}_{k,l}$$

Above all, we give the algorithm that generates the ε∼τ approximation of minimum AEs under the Lp constraint in Algorithm 1.

**Algorithm 1:** Algorithm of the generating ε∼τ approximation of minimum AEs under Lp constraint **Input:** a point $X_{0}\in R^{I_{1}\times I_{2}\times \ldots \times I_{N}},\varepsilon ∼\tau$ approximation of minimum adversarial    perturbations $\varepsilon_{\tau }^{*}$, a neural network *F*, the non-trivial lower bounds $\varepsilon_{NNS}$ of the    minimum adversarial perturbations of $X_{0}$ **Input: Parameters:** number of iterations *n*, *α* **Output:**
$X_{\tau }^{*}$
    1:**for***e* in *n* **do**    2: **if** *e* = 0 **then**    3:  *X* = *X*_0_    4:  $\Delta \varepsilon \;=\;\varepsilon_{NNS}$    5: **end if**    6: $\varpi_{i_{2},i_{2},\ldots ,i_{N}}\;=\;-\nabla_{X}\hbar (X)$    7: $\xi_{i_{2},i_{2},\ldots ,i_{N}}\;=\;\mathrm{DCT}{\left(X^{\Pi }\right)}_{k,l}$    8: $\Psi \;=\;\varpi_{i_{2},i_{2},\ldots ,i_{N}}\;+\;\alpha \xi_{i_{2},i_{2},\ldots ,i_{N}}\mathrm{DCT}{\left(X_{\Pi }\right)}_{k,l}$    9: $\Delta \varepsilon \;=\;(\varepsilon_{\tau }^{*}\;-\;\varepsilon_{NNS})/(n\;-\;1)$    10: X = X + Ψ × Δε    11:**end for**    12:return $X_{\tau }^{*}$


## 5. AEs Generation under SSIM Constraint

We model the problem of generating AEs under SSIM [17] measure metrics as follows. We use SSIM to replace the *D* measure metrics in Equation (8). For a neural network *F*, input distribution $ℵ\subset R^{n}$, a point $X_{0}\in ℵ$, the problem of generating controllable AEs of SSIM can be modeled as

(23) $$\begin{array}{c} F\left(X_{\tau }^{*}\right)\neq F\left(X_{0}\right)\\ \;s.t.\;X_{\tau }^{*}\in H_{p}=\left\{X:SSIM\left(X_{\tau }^{*},X_{0}\right)=\varepsilon_{\tau }^{*}\right\} \end{array}$$

According to the definition of the similarity measurement SSIM, for gray-scale images $x,y\in R^{n}$ as

(24) $$\mathrm{SSIM}\left(x,y\right)={\left[l\left(x,y\right)\right]}^{\varsigma }\cdot {\left[c\left(x,y\right)\right]}^{\theta }\cdot {\left[s\left(x,y\right)\right]}^{\iota }$$

where $l\left(x,y\right)=\frac{2\mu_{x}\mu_{y}+C_{1}}{\mu_{x}^{2}+\mu_{y}^{2}+C_{1}}$ defines the luminance, $c\left(x,y\right)=\frac{2\sigma_{x}\sigma_{y}+C_{2}}{\sigma_{x}^{2}+\sigma_{y}^{2}+C_{2}}$ defines the contrast comparison function, and $s\left(x,y\right)=\frac{\sigma_{xy}+C_{3}}{\sigma_{x}\sigma_{y}+C_{3}}$ defines the structure comparison function. Furthermore, $\mu_{x},\mu_{y}$ define the mean value of inputs x,y, respectively, $\sigma_{x},\sigma_{y}$ define the standard deviation of x,y, respectively, and $\sigma_{xy}$ is the covariance between *x* and *y*. $C_{1},C_{2},C_{3}>0$ and ς,θ,ι>0 are constants. According to [17], when setting ς=θ=ι=1 and $C_{3}=C_{2}/2$, Equation (24) can be simplified as,

(25) $$\mathrm{SSIM}\left(x,y\right)=\frac{\left(2\mu_{x}\mu_{y}+C_{1}\right)\left(2\sigma_{xy}+C_{2}\right)}{\left(\mu_{x}^{2}+\mu_{y}^{2}+C_{1}\right)\left(\sigma_{x}^{2}+\sigma_{y}^{2}+C_{2}\right)}$$

Furthermore, according to Lagrangian constraint, we formulate Equation (26) as

(26) $$L\left(X_{\tau }^{*},\varrho \right)=loss{\left(F\left(X_{\tau }^{*}\right),t\right)}^{2}+\varrho {\left(SSIM\left(X_{\tau }^{*},X_{0}\right)-\varepsilon_{\tau }^{*}\right)}^{2}$$

where ϱ is the Lagrangian valuable, *t* is the one-hot tensor of the target label and loss is the cross-entropy loss function as shown in Equation (27).

Cross-entropy can measure the difference between two different probability distributions in the same random variable. In machine learning, it is expressed as the difference between the target probability distribution *t* and the predicted probability distribution $F(X_{\tau }^{*})$.

(27) $$loss\left(F\left(X_{\tau }^{*}\right),t\right)=-\frac{1}{k}\sum_{i=1}^{k}\left[t_{i}\log F_{i}\left(X_{\tau }^{*}\right)+\left(1-t_{i}\right)\log\left(1-F_{i}\left(X_{\tau }^{*}\right)\right)\right]$$

## 6. Experimental Results and Discussion

### 6.1. Experimental Setting

**Dataset:** In this work, we evaluate our methods under two widely used datasets. MNIST is a handwriting digit recognition dataset from 0 to 9, including 70,000 gray images and 60,000 for training and 10,000 for testing. CIFAR-10 [32] has 60,000 images of ten classes, including airplane, automobile, bird, cat, deer, dog, frog, horse, ship and truck.

**Threat model:** In our paper, we generate the AEs of trained threat models. Due to limited computational resources, we train a feed-forward network with *p* layers and *q* neurons per layer. For all the networks, we use the ReLU activation function. We denote the networks as p×[q]. For the MNIST dataset, we train the 3×(1024) network as a threat model. For the CIFAR-10 dataset, we train 6×(1024), 7×(1024) and 6×[2048] as threat models.

**Baseline attack:** For comparing our method with other adversarial attacks, we generate AEs by different attack methods. Our method can adapt to different $L_{p}$ constraint measurements. In this part, due to limited computational resources, we adopt the $L_{2}$-constrained measurement. Therefore, we use other the $L_{2}$-constrained attack methods as the baseline, including SA-$L_{2}$ [10], FGSM-$L_{2}$ [11], BIM-$L_{2}$ [33], PGD-$L_{2}$ [14] and DF-$L_{2}$ [6]. We compare the performance of those attacks with our method under different $\varepsilon_{\tau }^{*}$ constraint.

### 6.2. Evaluation Results

#### 6.2.1. Results of Attack Ability

We calculate the success rates of the attacks to compare the attack ability. Due to the uncontrollable ability of the perturbations of other baseline attack, we first set the $\varepsilon_{\tau }^{*}$ as 0.4, 0.8 and 1.2 for the MNIST dataset and 20, 25, 30 and 37 for the CIFAR-10 dataset, and we obtain the average perturbations of the baseline attacks under the $L_{2}$ constraint, as shown in Table 1 and Table 2, and then we use their average perturbations as the $\varepsilon_{\tau }^{*}$ of our method under the same constraint and make a comparison of the success rates.

The criteria for selecting the values for the baseline for each dataset is that the value is sufficiently adequate for the baseline attack. This means that under that value, the baseline attack will not jump out of the circulation of attack in advance due to an excessively large value, which leads to the measured average perturbations not having enough correlation with that value. Meanwhile, that value will not lead to the low success rate of the baseline attack due to it being too small. Specifically, because the baseline attack cannot control the average perturbations, we first take the way of binary search that the range is (0,100] and the value interval is five and test the attack success rate and average perturbations of the baseline attack under different values. We then remove the points where either the difference between the average perturbations and that value is too large or the success rate is too low, that is, the points where that value overflows or is insufficient.

Due to the same average perturbations of the PGD-$L_{2}$ and BIM-$L_{2}$ attacks, we show their results in one table, namely Table 3 for MNIST and Table 4 for CIFAR. Furthermore, the comparison of the FGSM-$L_{2}$ attack and our method is shown in Table 5 for MNIST and Table 6 for CIFAR. As the four tables show, under the same $L_{2}\left(\varepsilon_{\tau }^{*}\right)$ constraint, our attack has a better attacking performance than other PGD-$L_{2}$($\varepsilon_{\tau }^{*}$), BIM-$L_{2}$($\varepsilon_{\tau }^{*}$) and FGSM-$L_{2}$($\varepsilon_{\tau }^{*}$) attacks.

In addition to the attacks that have a fixed $\varepsilon_{\tau }^{*}$, we also compare the attacks without a value to constrain the perturbations including the SA-$L_{2}$ and DF attacks. We also calculate the average perturbations of those attacks. Furthermore, then we use the same average perturbations as the $\varepsilon_{\tau }^{*}$ of our method and make a comparison in Table 7 and Table 8. For MNIST, our method has a better performance than the DF and SA attacks.

In addition to the above two small-sized datasets, the experiment also evaluates the performance of the algorithm on the larger and more complex dataset that is TinyImagenet. The dataset has 200 classes, each class has 500 pictures and we extract 200 pictures as the experimental data. For this dataset, we select the CNN model with seven layers that is denoted by ’CNN-7layer’ [34] as the threat model. Furthermore, we set the $\varepsilon_{\tau }^{*}$ as 1.0,2.0,4.0 and 6.0. The experiment first measures the average perturbation of the baseline attack under the selected $\varepsilon_{\tau }^{*}$. Furthermore, it then sets the average perturbation as the $\varepsilon_{\tau }^{*}$ to compare the success rate of our algorithm and the baseline attack under that same value. The average perturbation of the baseline attack is shown in Table 9 and the comparison of the attack ability is shown in Table 10. As shown in Table 10, our algorithm has a better performance than the FGSM attack under the same $\varepsilon_{\tau }^{*}$.

Furthermore, we also evaluate the attack ability of our algorithm on more complex models. We select Wide-ResNet, ResNeXt, and DenseNet as the target models and train them under the CIFAR dataset. The detail is the same as in [34]. The benchmark values we selected are 1.0,5.0,10.0,30.0,60.0 and 80.0. Similarly, we first calculate the average perturbations of the baseline attack under that values. Then, we evaluate the results of the success rate of our algorithm and the baseline attack under the same $\varepsilon_{\;tau}^{*}$ of our algorithm which is the same as the average perturbations calculated beforehand. Table 11 shows the average perturbations. We make a comparison of the attack ability in Table 12 and Due to Table 12, we find that under the benchmark values 5.0,10.0,30.0,60.0 and 80.0, our algorithm performs better than the FGSM attack. However, under the 1.0, in the Wide-ResNet and ResNeXt, the FGSM attack performs better.

#### 6.2.2. Results of SSIM Constraint under Different $\varepsilon_{\tau }^{*}$

**Figure 3:**
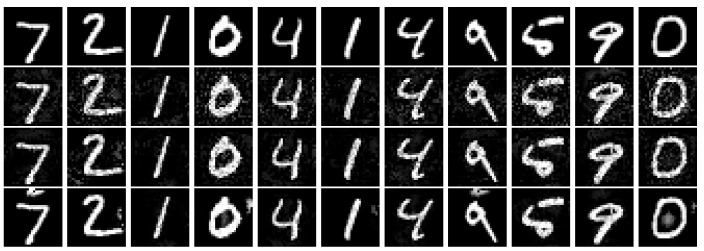
Figure of the images of the AEs generated under the SSIM constraint with different $\varepsilon_{\tau }^{*}$. The first line is the clean images and the second line shows the adversarial images under 0.5 constraint. The third line is the adversarial images under 0.7 constraint. The last line is the adversarial images under 0.9 constraint.

In this part, we evaluate our method described in Section 5. Due to there being no work devised for the same purpose as our method, we only show the results of our method without any comparison with others. We show the controllable ability under the SSIM constraint of our method and record its success rate in Table 13. We also show the adversarial images under different SSIM constraints in Figure 3.

#### 6.2.3. Results of α≠0 under $L_{2}$ Constraint

**Figure 4:**
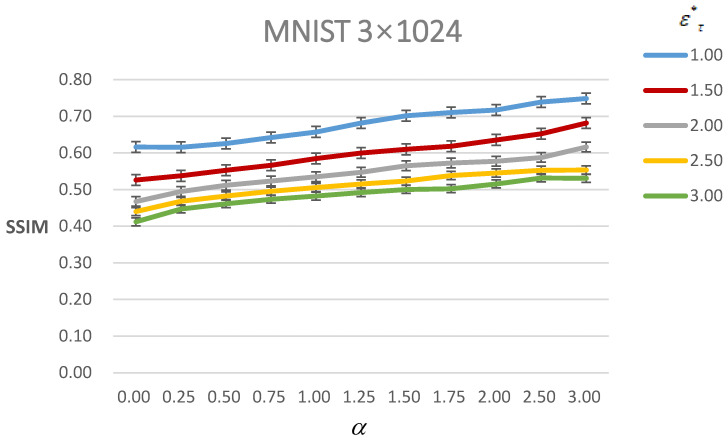
Figure of the SSIM value of AEs of MNIST under different $\varepsilon_{\tau }^{*}$ with different α. The line with different color means different $\varepsilon_{\tau }^{*}$.

In this section, we discuss the results of our method under $L_{2}$ with the α≠0 constraint. Through the different α, we can not only generate the controllable AEs but also improve the perceptual visual quality under the same $\varepsilon_{\tau }^{*}$ constraint. When the $\varepsilon_{\tau }^{*}$ under the $L_{2}$ constraint is large, the perceptual visual quality is poor. In order to adapt to this situation, our paper devises α to improve the perceptual visual quality. However, there is a trade-off between the visual quality of the AEs and their success rate. Figure 4 shows the SSIM value of AEs under different $\varepsilon_{\tau }^{*}$ with different α. As it shows, the SSIM value increases with the α increasing under the same $\varepsilon_{\tau }^{*}$ constraint. Furthermore, we can see that with the increasing $\varepsilon_{\tau }^{*}$, the SSIM value has a trend of decreasing under the same α. This means that the visual quality becomes poorer when more perturbations are added to the inputs, which is in line with the intuition of the AEs. Meanwhile, the SSIM value rises rapidly before α=1.0 under the same $\varepsilon_{\tau }^{*}$ constraint; after that, its trend tends to be flatter.

**Figure 5:**
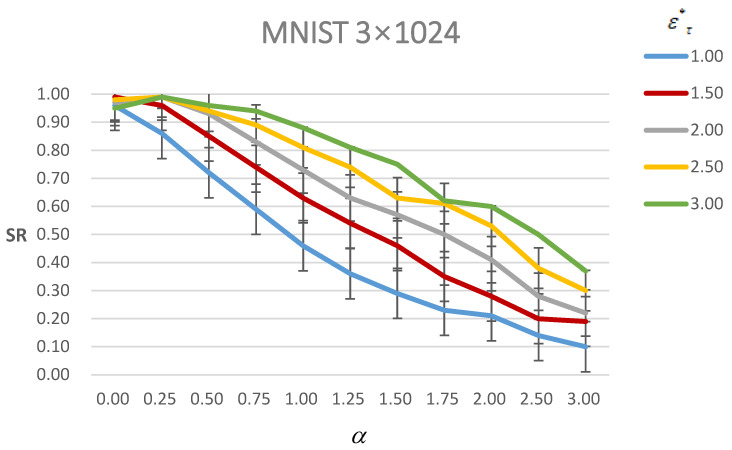
Figure of the success rate (SR) of AEs of MNIST under different $\varepsilon_{\tau }^{*}$ with different α. The line with a different color means different $\varepsilon_{\tau }^{*}$.

Figure 5 shows the success rate of AEs under different $\varepsilon_{\tau }^{*}$ with different α. As it shows, the success rate decreases with the α increasing under the same $\varepsilon_{\tau }^{*}$ constraint. Moreover, with the increasing $\varepsilon_{\tau }^{*}$, the success rate increases under the same α constraint. It is also consistent with the general nature of the AEs that when more perturbations are added, the probability of a successful attack becomes greater. Furthermore, the α=1.0 still tends to be a boundary that before α=1.0, the success rate decreases slower and then it decreases faster when $\varepsilon_{\tau }^{*}=3.00$ and $\varepsilon_{\tau }^{*}=2.50$. However, it has a nearly consistent trend of decreasing with $\varepsilon_{\tau }^{*}=1.00$, $\varepsilon_{\tau }^{*}=1.50$ and $\varepsilon_{\tau }^{*}=2.00$. It means that when the perturbations remain small, excessive attention to visual quality will lead to a greater loss of attack success rate. Therefore, it corresponds to the actual meaning of the parameter α that only needs to be set to α≠0 when $\varepsilon_{\tau }^{*}$ is large. We set α=1 and compare the results between α=0 and α=1 in Table 14.

**Figure 6:**
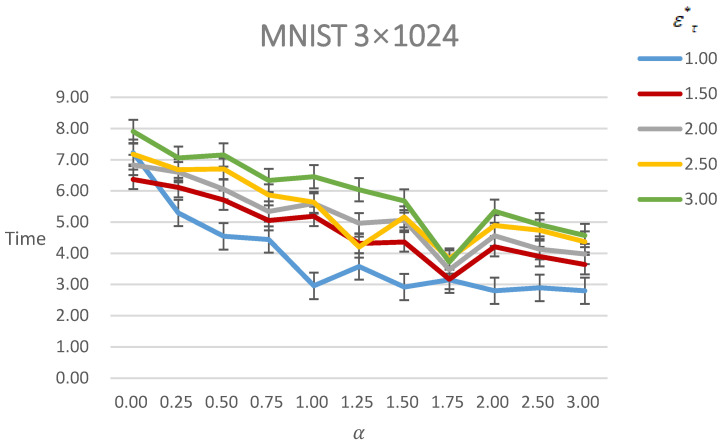
Figure of time of generating AEs of MNIST under different $\varepsilon_{\tau }^{*}$ with different α. The line with a different color means different $\varepsilon_{\tau }^{*}$.

Figure 6 shows the time of generating AEs under different $\varepsilon_{\tau }^{*}$ with different α. As it shows, the time decreases with the α increasing under the same $\varepsilon_{\tau }^{*}$ constraint. Moreover, with the $\varepsilon_{\tau }^{*}$ increasing, the time increases under the same α constraint.

## 7. Conclusions

Aiming at the two fundamental problems of generating the minimum AEs, we first define the concept of the minimum AEs and prove that generating the minimum AEs is an NPC problem. Based on this conclusion, we then establish a new third kind of optimization model that takes the successful attack as the target and the adversarial perturbations equal the lower bound of the minimum adversarial distortion plus a controllable approximation. This model generates the controllable approximation of the minimum AEs. We give a heuristic solution method of that model. From the theoretical analysis and experimental verification, our model’s AEs have a better attack ability and can generate more accurate and controllable AEs to adapt to different environmental settings. However, the method in this paper of the model does not perfectly determine the solution of the model, which will be the focus of future research.

## Figures and Tables

**Table 1:** Table of the average perturbations of different attack methods under MNIST. We compare our method with PGD-$L_{2}$, FGSM-$L_{2}$, BIM-$L_{2}$ attacks. We denote the feed-forward networks as p×[q] and *p* denotes the number of layers and *q* is the number of neurons per layer.

Model		$\varepsilon_{\tau }^{*}$	0.400	0.800	1.200	1.400
	Attack					
MNIST 3 × (1024)	PGD-$L_{2}$		0.399	0.799	1.199	1.399
	BIM-$L_{2}$		0.399	0.799	1.199	1.399
	FGSM-$L_{2}$		0.399	0.494	1.018	1.191

**Table 2:** Table of the average perturbations of different attack methods under CIFAR. We compare our method with PGD-$L_{2}$, FGSM-$L_{2}$, BIM-$L_{2}$ attacks. We denote the feed-forward networks as p×[q] and *p* denotes the number of layers and *q* is the number of neurons per layer.

Model		$\varepsilon_{\tau }^{*}$	20.000	25.000	30.000	37.000
	Attack					
CIFAR 6 × (1024)	PGD-$L_{2}$		19.573	24.062	28.070	32.630
	BIM-$L_{2}$		19.573	24.062	28.070	32.630
	FGSM-$L_{2}$		19.568	24.065	28.058	32.619
CIFAR 7 × (1024)	PGD-$L_{2}$		19.703	24.130	28.002	32.636
	BIM-$L_{2}$		19.703	24.130	28.002	32.636
	FGSM-$L_{2}$		19.703	24.123	27.993	32.624
CIFAR 6 × (2048)	PGD-$L_{2}$		19.776	24.347	28.598	33.613
	BIM-$L_{2}$		19.776	24.347	28.598	33.613
	FGSM-$L_{2}$		19.774	24.345	28.593	33.604

**Table 3:** Table of the success rate of PGD-$L_{2}$, BIM-$L_{2}$ and our $L_{2}$ attacks under MNIST. We denote the feed-forward networks as p×[q] whilst *p* denotes the number of layers and *q* is the number of neurons per layer.

Model		$\varepsilon_{\tau }^{*}$	0.399	0.799	1.199	1.399
	Attack					
MNIST 3 × (1024)	PGD-$L_{2}$		10.27	22.48	77.31	86.26
	BIM-$L_{2}$		10.27	39.36	77.51	86.57
	Our $L_{2}$		22.40	72.96	91.62	95.01

**Table 4:** Table of the success rate of PGD-$L_{2}$, BIM-$L_{2}$ and our $L_{2}$ attacks under CIFAR. We compare our method with PGD-$L_{2}$, FGSM-$L_{2}$, BIM-$L_{2}$ attacks. We denote the feed-forward networks as p×[q] and *p* denotes the number of layers and *q* is the number of neurons per layer.

Model		$\varepsilon_{\tau }^{*}$	19.573	24.062	28.070	32.630
	Attack					
CIFAR 6 × (1024)	PGD-$L_{2}$		17.36	24.86	31.99	38.39
	BIM-$L_{2}$		17.36	24.86	31.99	38.39
	Our $L_{2}$		66.80	69.20	72.80	79.00
CIFAR 7 × (1024)	PGD-$L_{2}$		22.28	28.10	32.40	39.22
	BIM-$L_{2}$		22.28	28.17	32.41	39.22
	Our $L_{2}$		83.60	88.00	94.00	90.20
CIFAR 6 × (2048)	PGD-$L_{2}$		18.19	27.38	34.45	41.34
	BIM-$L_{2}$		18.19	27.38	34.45	41.34
	Our $L_{2}$		73.60	78.00	81.60	86.80

**Table 5:** Table of the success rate of FGSM-$L_{2}$ and our $L_{2}$ attacks under MNIST. We denote the feed-forward networks as p×[q] and *p* denotes the number of layers and *q* is the number of neurons per layer.

Model		$\varepsilon_{\tau }^{*}$	0.399	0.494	1.018	1.191
	Attack					
MNIST 3 × (1024)	FGSM-$L_{2}$		7.12	39.60	49.54	61.85
	Our $L_{2}$		22.00	40.26	90.00	91.62

**Table 6:** Table of the success rate of PGD-$L_{2}$, BIM-$L_{2}$ and our $L_{2}$ attacks under CIFAR. We compare our method with PGD-$L_{2}$, FGSM-$L_{2}$, BIM-$L_{2}$ attacks. We denote the feed-forward networks as p×[q] and *p* denotes the number of layers and *q* is the number of neurons per layer.

Model		$\varepsilon_{\tau }^{*}$	19.568	24.065	28.058	32.619
	Attack					
CIFAR 6 × (1024)	FGSM-$L_{2}$		17.36	25.04	31.99	38.39
	Our $L_{2}$		66.80	69.30	72.80	79.00
Model		$\varepsilon_{\tau }^{*}$	19.703	24.123	27.993	32.624
	Attack					
CIFAR 7 × (1024)	FGSM-$L_{2}$		22.28	28.10	32.40	39.22
	Our $L_{2}$		83.60	88.00	94.00	90.20
Model		$\varepsilon_{\tau }^{*}$	19.774	24.345	28.593	33.604
	Attack					
CIFAR 6 × (2048)	FGSM-$L_{2}$		18.19	27.38	34.45	41.34
	Our $L_{2}$		73.60	78.00	81.60	86.80

**Table 7:** Table of the success rate of different attack methods under MNIST. We compare our method with SA-$L_{2}$ and DF attacks. We denote the feed-forward networks as p×[q] and *p* denotes the number of layers and *q* is the number of neurons per layer.

Model		Attacks	SA-$L_{2}$	Our $L_{2}$	DF	Our $L_{2}$
	Metrics					
MNIST 3 × (1024)	Average Perturbations		6.020	6.020	14.935	14.935
	Success Rate		99.89	100.00	100.00	100.00

**Table 8:** Table of the success rate of different attack methods under CIFAR. We compare our method with SA-$L_{2}$ and DF attacks. We denote the feed-forward networks as p×[q] and *p* denotes the number of layers and *q* is the number of neurons per layer.

Model		Attacks	SA-$L_{2}$	Our $L_{2}$	DF	Our $L_{2}$
	Metrics					
CIFAR 6 × (1024)	Average Perturbations		41.424	41.424	41.942	41.942
	Success Rate		80.80	85.28	95.61	95.90
CIFAR 7 × (1024)	Average Perturbations		47.846	47.846	60.050	60.050
	Success Rate		82.83	85.57	94.29	95.00
CIFAR 6 × (2048]	Average Perturbations		41.356	41.356	41.717	41.717
	Success Rate		81.45	85.00	96.81	97.28

**Table 9:** Table of the average perturbations of different attack methods under TinyImagenet. We compare our method with FGSM-$L_{2}$ attack. We use the feed-forward network cnn-7layer as the target model.

Model		$\varepsilon_{\tau }^{*}$	1.000	2.000	4.000	6.000
	Attack					
cnn-7layer	FGSM-$L_{2}$		0.999	1.999	3.999	5.999

**Table 10:** Table of the success rate of FGSM-$L_{2}$ and our $L_{2}$ attacks under TinyImagenet. We use the feed-forward network cnn-7layer as the target model.

Model		$\varepsilon_{\tau }^{*}$	0.999	1.999	3.999	5.999
	Attack					
cnn-7layer	FGSM-$L_{2}$		50.40	64.50	75.50	79.30
	Our $L_{2}$		22.00	55.50	80.50	88.00

**Table 11:** Table of the average perturbations of different attack methods under Cifar. We compare our method with FGSM-$L_{2}$ attack. We use the feed-forward networks Wide-ResNet, ResNeXt and DenseNet as the target models.

Model		$\varepsilon_{\tau }^{*}$	1.000	5.000	10.000	30.000	60.000	80.000
	Attack							
Wide-ResNet	FGSM-$L_{2}$		0.999	4.999	9.999	29.999	59.999	79.999
ResNeXt	FGSM-$L_{2}$		0.999	4.999	9.999	29.999	59.999	79.999
DenseNet	FGSM-$L_{2}$		0.999	4.999	9.999	29.999	59.999	79.999

**Table 12:** Table of the success rate of FGSM-$L_{2}$ and our $L_{2}$ attacks under Cifar. We use the feed-forward networks Wide-ResNet, ResNeXt and DenseNet as the target models.

Model		$\varepsilon_{\tau }^{*}$	0.999	4.999	9.999	29.999	59.999	79.999
	Attack							
Wide-ResNet	FGSM-$L_{2}$		28.50	44.50	51.00	68.00	87.50	87.00
	Our $L_{2}$		25.00	47.50	66.00	90.50	99.50	100.00
ResNeXt	FGSM-$L_{2}$		23.50	30.50	34.00	51.50	67.50	74.00
	Our $L_{2}$		21.50	41.00	51.50	87.50	93.50	90.00
DenseNet	FGSM-$L_{2}$		25.50	33.00	36.00	43.50	53.50	55.50
	Our $L_{2}$		30.00	38.50	51.00	70.00	77.00	77.00

**Table 13:** Table of the controllable ability and attack ability of our method under the SSIM constraint. The perturbation coefficient of the attack is marked in brackets as SSIM-$\varepsilon_{\tau }^{*}$. We denote the feed-forward networks as p×[q] and *p* denotes the number of layers and *q* is the number of neurons per layer.

	Dataset	MNIST 3 × (1024)		CIFAR 6 × (1024)		CIFAR 7 × (1024)		CIFAR 6 × (2048)	
Attack		SSIM	SR	SSIM	SR	SSIM	SR	SSIM	SR
Our SSIM(0.5)		0.500	100.00	0.500	100.00	0.500	100.00	0.500	100.00
Our SSIM(0.7)		0.700	96.60	0.700	100.00	0.700	100.00	0.700	100.00
Our SSIM(0.9)		0.900	42.00	0.900	31.00	0.900	36.50	0.900	36.00

**Table 14:** Table for the AEs under α=0 and α=1 of the $L_{2}$ constraint. We denote the feed-forward networks as p×[q] and *p* denotes the number of layers and *q* is the number of neurons per layer. The AP, SR, SSIM and Time denote the average perturbations, the success rate, the SSIM value between the original image and adversarial image and the time taken to generate AEs, respectively.

Model		Attack	$\varepsilon_{\tau }^{*}$ = 0.50		$\varepsilon_{\tau }^{*}$ = 1.00		$\varepsilon_{\tau }^{*}$ = 1.20		$\varepsilon_{\tau }^{*}$ = 2.40	
	Metrics		α = 0	α = 1	α = 0	α = 1	α = 0	α = 1	α = 0	α = 1
MNIST 3 × (1024)	AP		0.50	0.50	1.00	1.00	1.20	1.20	2.40	2.40
	SR		38.71	12.9	85.16	43.5	91.62	51.9	98.19	74.7
	SSIM		0.77	0.82	0.61	0.67	0.57	0.62	0.42	0.52
	Time		8.33	9.33	7.22	9.19	6.82	9.34	7.07	9.31

**Figure A1:**
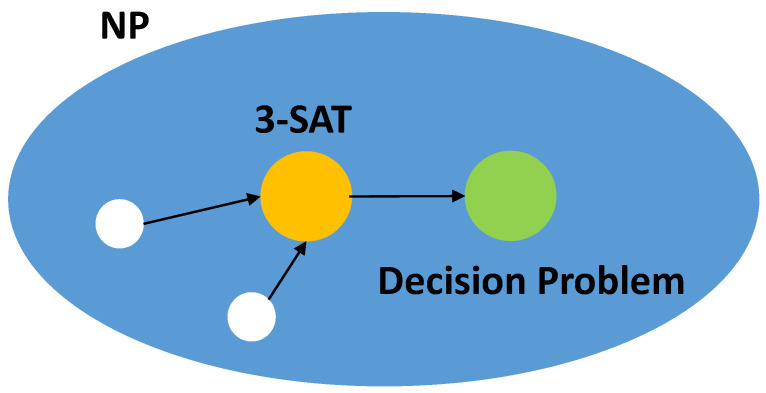
Figure for the transitivity of polynomial simplification.

**Figure A2:**
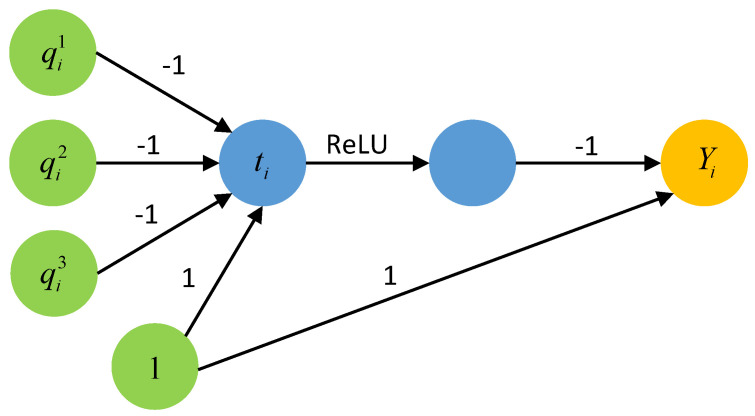
Figure for the disjunctive gadget when $q_{i}$ are variables.

**Figure A3:**
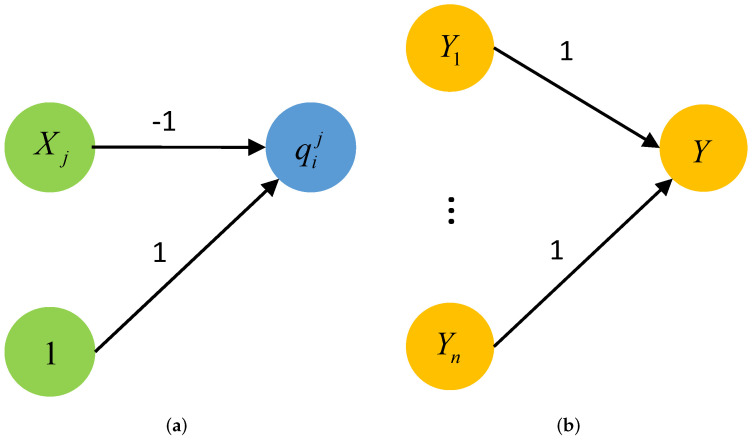
Figure for negative disjunction and conjunction gadgets. (**a**) Figure representing a negative disjunction gadget. (**b**) Figure representing a conjunction gadget.

**Figure A4:**
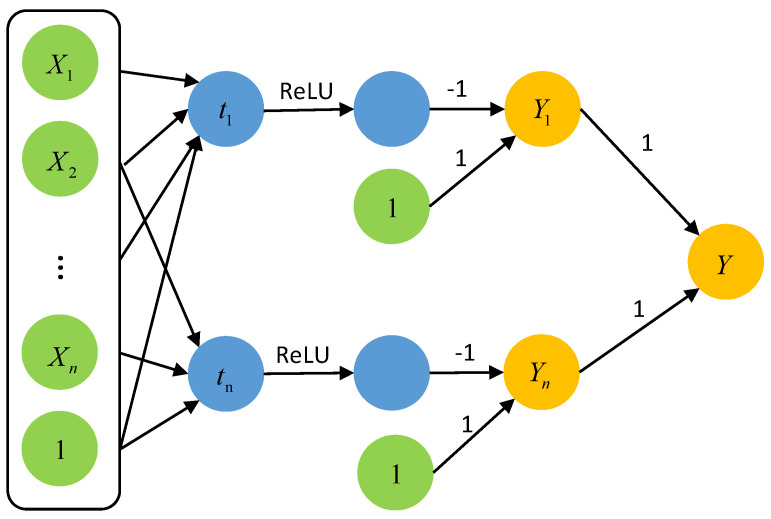
Figure for the 3-SAT-DNN conjunction gadget.

## Data Availability

Not applicable.

## Appendix A

### Appendix A.1. Proof of NPC of Calculating the Minimum Adversarial Perturbations

**Definition** **A1.**
*(3-SAT). Given a finite set of Boolean variables $B=B_{1},B_{2},\ldots B_{n}$, |B|=n, each variable takes 0 or 1, and a set of clauses $C=C_{1},C_{2},\ldots C_{k}$, |C|=k, $\psi =C_{1}\wedge C_{2}\wedge \ldots \wedge C_{k}$; each $C_{i}$ is a disjunctive normal form composed of three variables, that is, $Z_{1}\vee Z_{2}\vee Z_{3}$. Question: given a Boolean variable set X and clause set C, whether there is a true value assignment so that C is true and then each clause is true.*

**Theorem** **A1.**
*Given a neural network F, a distribution $ℵ\subset R^{n}$, and a distance measurement $D:R^{n}\times R^{n}\to R$ between X and $X_{0}$, a point $X_{0}\in R^{n}$, searching for a minimum adversarial example of $X_{0}$ is an NPC problem.*

**Proof.** We now prove Theorem A1. We first reduce the problem into a decision problem, and then according to the definition of an NPC problem, we prove that the problem belongs to the type of NP problem, and finally, we prove that a known NPC problem can be reduced to the decision problem in polynomial time. We first reduce the problem of finding the minimum AEs into a series of decision problems. Though many important problems are not decision problems when they appear in the most natural form, they can be reduced to a series of decision problems that are easier to study, for example, the coloring problem of a graph. When coloring the vertices of a graph, we need at least *n* colors to make any two adjacent vertices have different colors. Then, it can be transformed into another question. Can we color the vertices of the graph with no more than *m* colors, m∈N+? The first *m* value in the set that makes the problem solvable is the optimal solution of the coloring problem. Similarly, we can also transform the optimization problem of finding the minimum AEs into the following series of decision problems: given the precision of perturbations δε, and initial perturbations $\varepsilon_{1}$, $\varepsilon_{i}=\varepsilon_{i-1}+\delta \varepsilon$, whether we can use the perturbations ε, $\varepsilon \in \varepsilon_{1},\varepsilon_{2},...,\varepsilon_{i},...$ to make the inequality $F\left(X_{0}{+}^{*}\varepsilon_{i}\right)\neq F\left(X_{0}\right))$ true, and the first value in the sequence that makes the inequality true is the optimal solution of the optimization problem. The decision problem is reduced and formalized as follows. For the neural network attribute, $\varphi =\varphi_{1}\left(X\right)\wedge \varphi_{2}\left(Y\right)$, $\varphi_{1}$ is the mapping from the AEs generated by the AEs generation function to label 1, that is, $\varphi_{1}\left(X\right):\left(X{+}^{*}\varepsilon \right)\to 1$. $\varphi_{2}$ is the mapping from the AEs generated by the AEs generation function to 0,1, that is, $\varphi_{2}\left(X\right):\left(X{+}^{*}\varepsilon \right)\to 0,1$. When $F\left(X{+}^{*}\varepsilon \right)\neq F\left(X\right)$, its value is 1. When $F\left(X{+}^{*}\varepsilon \right)=F\left(X\right)$, its value is 0. When there is an assignment $\alpha \left(X\right)=X{+}^{*}\varepsilon_{i}$, $\varepsilon \in \varepsilon_{1},\varepsilon_{2},...,\varepsilon_{i},...$, α(Y) is the output of the neural network and determines whether the value of the attribute φ is true. Obviously, it is an NP problem. In the guessing stage, given any perturbations ε, assuming the ε is a candidate solution of the decision problem. Furthermore, then in the verification stage, since the process of inputting perturbations ε and samples $X_{0}$ to the neural network and then outputting the results can be completed in polynomial time, it is polynomial in the verification stage. Therefore, the solution to the decision problem is an uncertain polynomial algorithm. Furthermore, according to the definition of the NP problem, the decision problem is an NP problem. Finally, we prove that any problem in NP can be reduced to the decision problem in polynomial time. Due to the transitivity of polynomial simplification, we can prove a known NPC problem: the 3−SAT problem can be transformed into the decision problem in polynomial time and then complete this proof. Since the 3−SAT problem is an NPC problem, according to the definition of an NPC problem—that is, any problem in the set of NP problems that can be reduced to an 3−SAT problem in polynomial time—if the 3−SAT problem can be reduced to the aforementioned decision problem of searching for the AEs, according to transitivity, any problem in NP can be reduced to that decision problem in polynomial time and it can be proven that the decision problem is an NP-hard problem. We then prove how the 3−SAT problem is Turing reduced to the decision problem. According to the definition of an 3−SAT problem, given the Ternary satisfiability formula $\psi =C_{1}\wedge C_{2}\wedge \ldots \wedge C_{k}$ on the variable set $X=\left\{x_{1},x_{2},\ldots ,x_{k}\right\}$, each clause is a disjunction of three terms: $q_{i}^{1}\vee q_{i}^{2}\vee q_{i}^{3}$, where $q_{i}^{1}$, $q_{i}^{2}$ and $q_{i}^{3}$ are variables from *X* or their negative values. The problem is turned into that determining whether there is an assignment α:X→{0,1} to satisfy ψ, that is, whether there is an assignment α that makes all clauses $C_{i}$ valid at the same time. To simplify, we first assume that the input node $q_{i}^{1}$, $q_{i}^{2}$ and $q_{i}^{3}$ is a sub-statement constructed when the discrete value is 0 or 1. Then, we will explain how to relax this restriction so that the only restriction on the input nodes is that they are in the range of [0,1]. Firstly, we introduce the disjunctive tool, that is, given nodes $q_{1},q_{2},q_{3}\in 0,1$ and the output node is $Y_{i}$. When $q_{1}+q_{2}+q_{3}\geq 1$, $Y_{i}=1$, otherwise $Y_{i}=0$. The following Figure A2 shows the situation when $q_{i}$ is the variable itself (that is, it is not the negative value of the variable). The disjunctive tool can be seen as the process of calculating Equation (A1):

(A1) $$Y_{i}=1-\max\left(0,1-\sum_{j=1}^{3}q_{i}^{j}\right)$$

If it has one variable of input which is at least 1, then $Y_{i}=1$. If all the variables of input are 0, then $Y_{i}=0$. The key of the tool is that the ReLU function can ensure that the output $Y_{i}$ remains exactly 1 even if multiple inputs are set to 1. For processing any negative item $q_{i}^{j}=1-x_{j}\equiv \neg x_{j}$, we introduce a negative tool before inputting the negative item into the disjunctive tool, as shown in Figure A3a. The tool that calculates $1-x_{j}$ and then continues to calculate is the aforementioned disjunctive tool. The last step involves a conjunction widget, as shown in Figure A3b. Assuming that all nodes $Y_{1},\ldots ,Y_{n}$ are in the range of 0,1, we require node *Y* in the range of [n,n]. Obviously, this requirement only holds if all nodes are 1. Lastly, in order to check if all the clauses $C_{1},\ldots ,C_{n}$ are satisfied at the same time, we construct a conjunction gadget(using the negative value tool as input as needed) and combine it with a conjunction gadget, as shown in Figure A4. The input variable is mapped to each $t_{i}$ node according to the definition of clause $C_{i}$, that is, $t_{i}\to C_{i}$. According the above discussion, if the clause $C_{i}$ is satisfied, then $Y_{i}=1$; otherwise, $Y_{i}=0$. Therefore, the node *Y* is the range of [n,n] if and only if all the clauses are satisfied at the same time. Thus, an assignment α:X→0,1 of input satisfies the constraint between the input and the output of neural networks if and only if that assignment also satisfies the original item $\psi =C_{1}\wedge C_{2}\wedge \ldots \wedge C_{n}$. The above construction is based on the assumption that the input node takes values from discrete values 0,1, that is, α:X→0,1. However, it does not accord with the assumption that $\psi_{1}\left(X\right)$ is the conjunction of linear constraints. We will then prove how to relax the restriction to make the original proposition true. Letting ε is a very small number. We suppose that each variable $X_{i}$ is in the range of [0,1] but ensure that any feasible solution satisfies X∈[0,ε] or X∈[1−ε,1]. We add an auxiliary gadget to each input variable $X_{i}$, that is, using the ReLU function node to calculate Equation (A2) as follows:

(A2) max(0,ϵ−X)+max(0,X−1+ϵ)

Furthermore, the output node of Equation (A2) is required to be within the range [0,ε]. This expression can directly indicate that when X∈[0,ε] or X∈[1−ε,1], it is true for X∈[0,1]. The disjunctive expression in our construction is Equation (A1). The value of its disjunctive expression changes with the inputs. If all inputs are in [0,ε] or [1−ε,1], then at least one input is in [1−ε,ε] and then the end output node of each disjunctive gadget $Y_{i}$ will no longer use discrete values [0,1] but will be in [0,3ε]. If at least one node of each input clause is in the range [1−ε,1], then all $Y_{i}$ nodes will be in [1−ε,1] and *Y* will be in [n(1−ε),n]. However, if at least one clause does not have a node in the range [1−ε,1], *Y* will be less than n(1−ε) (when $\epsilon <\frac{1}{n+3}$). Therefore, keeping the requirements Y∈[n(1−ϵ),n] true, if and only if ψ is satisfied, its input and output will be satisfied, and the satisfied assignment can be constructed by making each $X_{i}\in \left[0,\varepsilon \right]=0$ and each $X_{i}\in \left[1-\varepsilon ,1\right]=1$. □

### Appendix A.2. Analysis of SSIM Constraint Method

We also try to directly calculate the adversarial perturbations as the $L_{p}$ constraint of our method. However, we find that it is difficult to perform the same operation under the SSIM constraint. The analysis is as follows. According to Equation (25) and substituting its inputs *x* and *y* as $X_{\varepsilon_{\tau }^{*}}^{*}$ and $X_{0}$, respectively, Equation (25) can be seen as the product of two parts [35]. That is:

(A3) $$\mathrm{SSIM}\left(X_{\varepsilon_{\tau }^{*}}^{*},X_{0}\right)=S_{1}\left(X_{\varepsilon_{\tau }^{*}}^{*},X_{0}\right)S_{2}\left(X_{\varepsilon_{\tau }^{*}}^{*},X_{0}\right)$$

where:

(A4) $$\begin{array}{cc} \; & S_{1}\left(X_{\varepsilon_{\tau }^{*}}^{*},X_{0}\right)=\frac{2\mu_{X_{\varepsilon_{\tau }^{*}}^{*}}\mu_{X_{0}}+C_{1}}{\mu_{X_{\varepsilon_{\tau }^{*}}^{*}}^{2}+\mu_{X_{0}}^{2}+C_{1}}=f\left(\mu_{X_{\varepsilon_{\tau }^{*}}^{*}},\mu_{X_{0}}\right)\\ \; & S_{2}\left(X_{\varepsilon_{\tau }^{*}}^{*},X_{0}\right)=c\left(X_{\varepsilon_{\tau }^{*}}^{*},X_{0}\right)s\left(X_{\varepsilon_{\tau }^{*}}^{*},X_{0}\right)=\frac{2\sigma_{X_{\varepsilon_{\tau }^{*}}^{*}X_{0}}+C_{2}}{\sigma_{X_{\varepsilon_{\tau }^{*}}^{*}}^{2}+\sigma_{X_{0}}^{2}+C_{2}}=g\left(X_{\varepsilon_{\tau }^{*}}^{*}-\mu_{X_{\varepsilon_{\tau }^{*}}^{*}},X_{0}-\mu_{X_{0}}\right) \end{array}$$

Therefore, the $\mathrm{SSIM}(X_{\varepsilon_{\tau }^{*}}^{*},X_{0})$ can be divided into the *f* function of $\mu_{X_{\varepsilon_{\tau }^{*}}^{*}}$ and $\mu_{X_{0}}$ and the *g* function of $X_{\varepsilon_{\tau }^{*}}^{*}-\mu_{X_{\varepsilon_{\tau }^{*}}^{*}}$ and $X_{0}-\mu_{X_{0}}$. In order to solve the condition of Equation (23), i.e., the product of the two functions needs to be a constant, we try to transform prime factorization to decompose *d* into the product of two values. Furthermore, the input $X_{0}$ is given so a set of prime factorization can be seen as solving a $X_{\varepsilon_{\tau }^{*}}^{*}$ that meets the criteria of Equation (A3).

However, the solutions $X_{\varepsilon_{\tau }^{*}}^{*}$ of the criteria of Equation (23) are not certain whether they are the AEs of the model *F*. Moreover, the solution of prime factorization is limited and it is a small set that meets the constraints, so it is more difficult to find AEs in that smaller set.

### Appendix A.3. The Definition of the Lower and Upper Bound of a Network

We recall this definition from [23] as follows:

**Definition** **A2.**
*(lower bound $\gamma_{y}^{L}$, upper bound $\gamma_{y}^{U}$). Given a neural network F, a distribution $ℵ\subset R^{n}$, a point $X_{0}\in R^{n}$, a label y, and the output of the network under y label $F_{y}$, we say that $\gamma_{y}^{L}$ and $\gamma_{y}^{U}$ are the lower bound and the upper bound of the network F under the label y such that $\gamma_{y}^{L}\leq F_{y}\left(X_{0}\right)\leq \gamma_{y}^{U}$.*

According to Definition A2, we give a further explanation of Equation (5). Give a point $X_{0}$ and a perturbation ε when inputting the perturbed point *X* of $X_{0}$ under ε, if the upper bound of the network under the original label *y* is lower than the lower bound of the network under the label $y^{*}\neq y$, meaning that the output $F_{y}\left(X\right)\leq F_{y}^{*}\left(X\right)$ so that *X* is an adversarial example.
